# What makes a scent trigger a memory? A cognitive decomposition of odor-evoked retrieval

**DOI:** 10.1016/j.isci.2025.114467

**Published:** 2025-12-17

**Authors:** Juliette Greco-Vuilloud, Perrine Ruby, Jane Plailly, Anne-Lise Saive

**Affiliations:** 1 Lyfe Research and Innovation Center, Ecully, France; 2 L'Oréal Research & Innovation, Clichy, France; 3 Perception, Attention, Memory Team, Lyon Neuroscience Research Center, CNRS UMR 5292 - INSERM U1028 - University Lyon1, 69366 Lyon, France; 4 Olfaction: from Coding to Memory Team, Lyon Neuroscience Research Center, CNRS UMR 5292 - INSERM U1028 - University Lyon1, 69366 Lyon, France

**Keywords:** Neuroscience, Cognitive neuroscience, Machine learning

## Abstract

A single scent can unlock vivid memories. This study investigates the factors that make some odors more evocative than others. We examined odor-evoked episodic memory in 106 participants who experienced odors embedded in distinct visuospatial contexts, and whose memory was tested 24–72 h later. The protocol empirically dissociates odor recognition (“I’ve already smelled this scent”) and associative memory (“It evokes a memory”) processes. Using machine learning with SHapley Additive exPlanations, we identified distinct predictors for each process. Recognition was driven by emotional strength, especially for unpleasant odors, and the richness of verbal descriptions. Associative memory followed a U-shaped relationship with familiarity and was strongly influenced by semantic distinctiveness—how uniquely each odor was described. Together, these findings reveal that odor memorability depends not only on its emotional salience but also on how specifically it is conceptualized and how familiar we are with it.

## Highlights

• Odor-evoked memory unfolds into recognition and associative retrieval
• Odor recognition is driven by unpleasant emotional strength
• Associative retrieval shows a U-shape relationship with odor familiarity
• Semantic richness and uniqueness of descriptions enhance odor-evoked memory

## Graphical abstract

**Figure:**
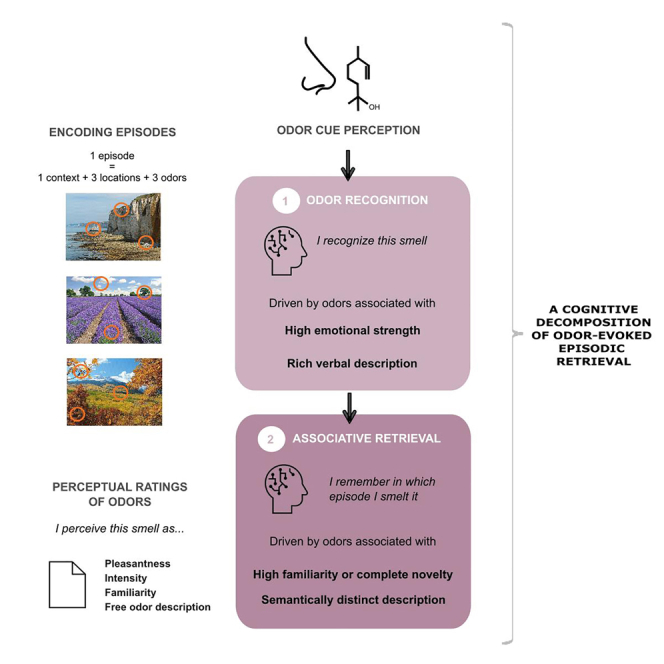

## Introduction

Odors stand out among sensory cues for their capacity to evoke vivid, emotional memories that often date back decades.^1^^,^^2^^,^^3^^,^^4^ Their privileged mnemonic power is commonly attributed to the direct anatomical connections that link the primary olfactory (piriform) cortex to the limbic structures such as the amygdala and hippocampus, bypassing the thalamic relay that gates every other sensory modality.^5^^,^^6^^,^^7^^,^^8^^,^^9^ Nevertheless, the specific perceptual and semantic factors that decide whether an odor will later prove memorable remain poorly understood.

Odor-evoked episodic memory unfolds in two stages: (1) recognizing that an odor has been encountered before and (2) retrieving the episode associated with it. Hereafter, we refer to these as odor recognition and odor associative retrieval. Odor pleasantness, emotional strength, intensity, familiarity, and semantic knowledge have all been reported as modulators of these stages, but the direction and magnitude of the reported effects vary widely across studies.^10^^,^^11^^,^^12^^,^^13^^,^^14^^,^^15^^,^^16^^,^^17^^,^^18^^,^^19^^,^^20^^,^^21^^,^^22^ Inconsistent findings likely stem from confounding recognition and associative recall as a single memory variable, overlooking the possibility that distinct odor attributes govern the two subprocesses.

Adding to the complexity is the exceptional inter-individual variability of human olfaction. Genetic polymorphism in the genes encoding olfactory receptors significantly influences the perceived intensity and pleasantness of an odor, thereby modulating the olfactory experience.^23^^,^^24^ On top of this genetic mosaic, other factors such as hormones, gender, age, and cultural background layer further heterogeneity in odor perception.^24^^,^^25^^,^^26^^,^^27^^,^^28^^,^^29^ Consequently, the statistical weight of any given odor attribute may be obscured unless models explicitly accommodate participant-specific variability.

One promising approach to addressing the variability and complexity in olfactory data is the use of artificial intelligence, which excels at identifying patterns in multidimensional datasets.^30^ For example, a neural network model was trained to decode the combinatorial code of 6,000 odorant molecules across 400 olfactory receptors, achieving performance equivalent to that of laboratory tests.^31^^,^^32^ Machine learning models also successfully predicted individual and population-level perceptions of odor intensity, pleasantness, and semantic descriptors based on the chemical characteristics of odorant molecules.^33^^,^^34^^,^^35^ Recently, a neural network-based model was developed to predict the odor quality of previously uncharacterized molecules from their chemical characteristics, achieving performance comparable to a panel of human experts.^36^ However, applying these models to smaller datasets, containing highly variable data, such as psychophysical and physiological features, limits model performance.^37^ Despite these challenges, integrating subjective dimensions and individual differences is essential for a comprehensive understanding of olfactory mechanisms and for developing approaches in human olfaction research that are more robust, and more resistant to bias.^38^^,^^39^

**Figure 1:**
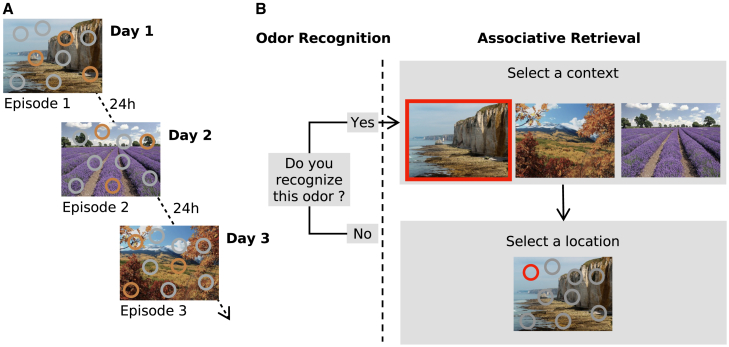
Experimental task design (A) Time course of encoding sessions. Participants experienced one episode per day over 3 days. Each episode consisted of a landscape picture in which, while gray circles were inactive, each orange circle symbolized spatial location of a particular odor which was delivered when the circle was clicked. (B) Example of a retrieval trial, on day 4. Each trial began with an odor recognition task. Upon answering “Yes” to recognizing an odor, participants proceeded to the associative part of the episodic memory retrieval task and described the context in which this odor had been previously encountered, by first selecting a context (from the three pictures) and then selecting a location (among the nine circles).

To clarify which odor attributes impact odor-evoked episodic memory, we disentangled odor recognition and odor associative retrieval subprocesses in a controlled paradigm (Figure 1F),^11^^,^^16^^,^^40^ and applied interpretable machine-learning models (SHapley Additive exPlanations)^41^ to identify distinct predictors for each subprocess, while explicitly accounting for the high individual variability that characterizes human olfaction.

## Results

### High individual differences in odor perception

Participants correctly recognized 77.49% ± 16.98 (mean ± standard deviation [SD]) of target odors (chance = 50%). Among recognized odors (hits), participants retrieved associated episodic details in 55.06% ± 22.39 of cases (chance = 33%).

Odors were rated as neutral in pleasantness (−0.12 ± 1.12 for targets and −0.19 ± 1.23 for hit odors), with moderate emotional strength (2.08 ± 0.75 for targets and 2.14 ± 0.82 for hit odors), moderate intensity (5.83 ± 1.28 for targets and 6.00 ± 1.33 for hit odors), moderate familiarity (4.86 ± 1.49 for targets and 4.93 ± 1.53 for hit odors), and described using relatively few words (1.50 ± 1.05 for targets and 1.57 ± 1.15 for hit odors) (Figures S1 and 2).

**Figure 2:**
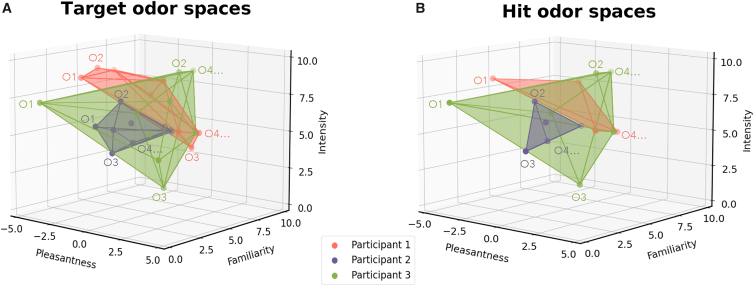
Participants represent odors in distinct perceptual spaces Perceptual spaces of three participants computed (A) from all target odors for the recognition component and (B) from all hit odors for the associative component of odor-evoked memory. Each odor, evaluated by the participants in terms of pleasantness, familiarity, and intensity, is represented by a point in a three-dimensional space. The convex envelope (enveloping volume) around the points indicates the distribution of odors in each participant’s perceptual space. The size and shape of the envelope reflect the variability of participants’ perceptual evaluations of odors.

To assess perceptual interindividual variability, we computed the SD of ratings for each odor, averaged these SDs across all odors for each perceptual dimension and compared them to the theoretical maximum SD defined as the SD obtained when ratings are split evenly between the minimum and maximum values (i.e., 5 for pleasantness, intensity, and familiarity; 2.5 for emotional strength; Table S1; Figure S2). Variability ranged from 45% (i.e., corresponding to a variability of ∼2.25 points on a 0–10 scale) to 61% (i.e., corresponding to a variability of ∼1.53 points on a 0–5 scale) of the theoretical maximum, depending on the dimension. These findings highlight the diversity in how odors were perceived across participants.

### Odor recognition is driven by emotional responses and richness of verbal descriptions

**Figure 3:**
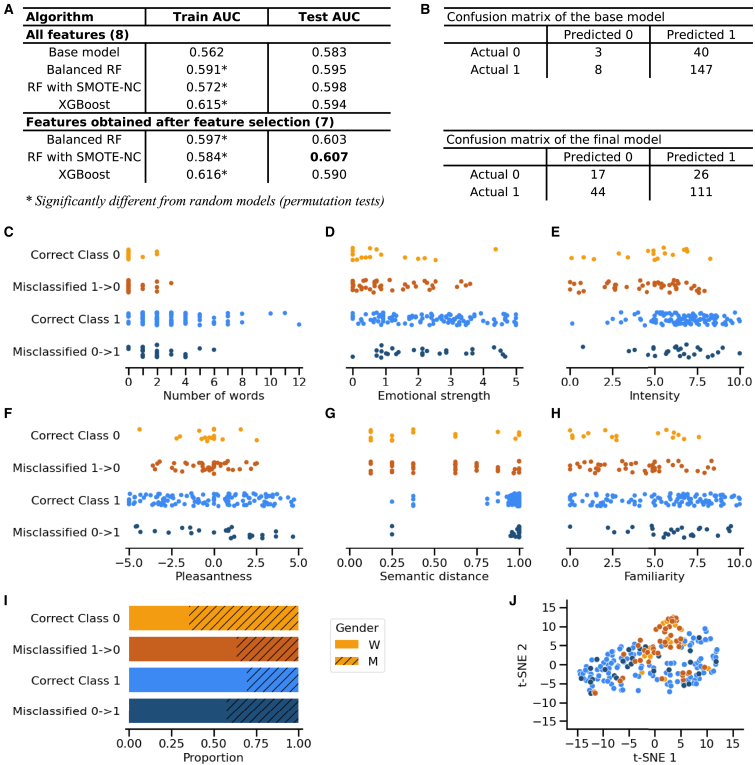
Errors in odor recognition reflect feature characteristics (A) AUC scores of different models during training and testing. (B) Confusion matrix showing the distribution of actual versus predicted odor recognition outcomes on the test set for both the base model and the final model. The final model improved classification performance for class 0. (C–H) Scatterplots showing individual test trials according to odor-related features: (C) intensity, (D) emotional strength, (E) pleasantness, (F) familiarity, (G) number of words in the description, (H) gender distribution per trial category, and (I) semantic distance between odors. (J) Dimensionality reduction of the seven features using t-SNE. These scatterplots illustrate the feature value ranges and their overlap between correctly and incorrectly classified trials: the feature value ranges of hits incorrectly identified as misses is close to those of correctly identified misses and the feature value ranges of misses incorrectly identified as hits is close to those of correctly identified hits. Correct class 0: correctly identified as miss (*n* = 17 trials); correct class 1: correctly identified as hit (*n* = 111); misclassified 0–>1: miss incorrectly identified as hit (*n* = 26); misclassified 1–>0: hit incorrectly identified as miss (*n* = 44).

The objective of this first analysis was to predict the odor recognition component. The best model was a random forest used after SMOTE-NC to address class imbalance. The random forest consisted of 2,000 trees, each with a maximum depth of 3 and up to 300 leaf nodes. It required a minimum of four samples per leaf and 20 samples for each split. The model also used a random state of 42 to ensure reproducibility of results. The final model included seven predictive features, from the most to the least important: intensity, emotional strength, pleasantness, familiarity, number of words, gender, and semantic distance. Permutation tests confirmed that the final model performed significantly better than chance (*p* = 0.001), where the baseline model did not (Figure S3). Examination of confusion matrices and test set scores confirmed the superiority of the optimized model (Figures 3A and 3B).

To better understand model errors, misclassified trials were analyzed and compared to correctly classified ones (Figures 3C–3J). We visualized these differences using scatterplots showing the ranges of feature values between correctly and incorrectly classified samples. These comparisons revealed that misclassifications tended to occur when feature value ranges overlapped between classes. Correctly predicted hits were associated with longer descriptions (up to 12 words), higher emotional strength (up to 5), higher intensity values (up to 10), greater pleasantness range ratings (−10 to 10), higher semantic distance between odors (>0.25), and higher familiarity ratings (up to 10). In contrast, misses were characterized by shorter descriptions (<3 words), lower emotional strength (<4), lower intensity values (<8), neutral pleasantness ratings (−3 to 3), lower semantic distance between odors (from 0.1), and lower familiarity (<8). Gender differences were also observed with hits more frequently predicted for women (30% M and 70% W), while misses were more often predicted for men (65% M and 35% W). Misclassifications reflected the characteristics of their assigned classes. Hits classified as misses resembled correctly classified misses and were predominantly made by women (35% M and 65% W), whereas misses classified as hits mirrored correctly classified hits and showed no clear gender bias (40% M and 60% W). A t-SNE projection in two-dimensional space further confirmed that errors clustered closely with correctly classified trials, suggesting that misclassifications reflected feature distributions similar to their predicted classes (Figure 3J).

**Figure 4:**
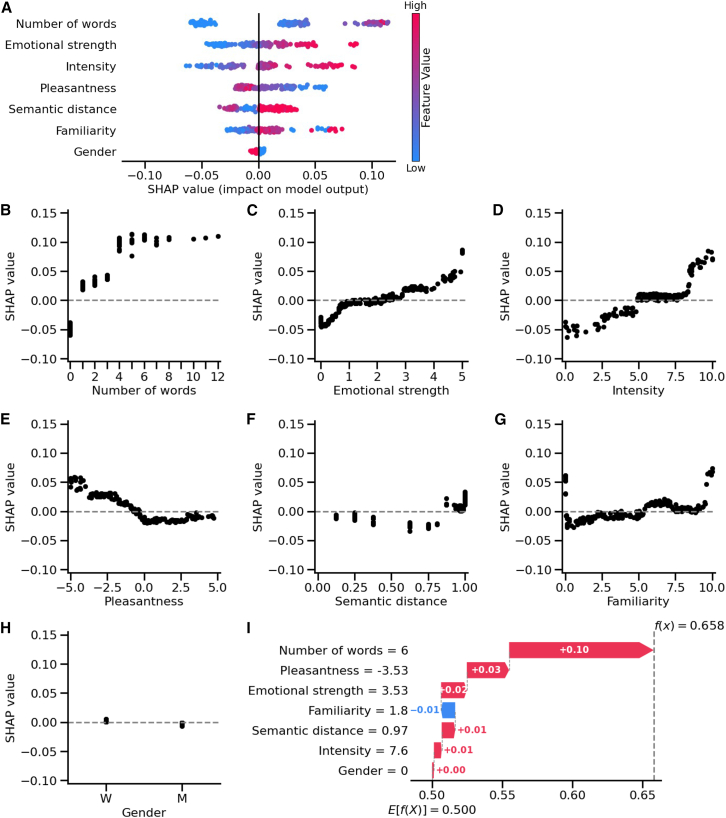
Odor recognition is primarily driven by emotional intensity and verbal richness (A) SHAP summary plot showing the feature contributions to odor recognition predictions. Each point represents an SHAP value for a single trial, with color indicating the feature’s value (blue for low and pink for high). For the gender feature, blue corresponds to female and pink to male. Positive SHAP values shift the prediction toward correct recognition, while negative SHAP toward incorrect recognition. (B–H) SHAP dependence plots illustrating the influence of individual features on the model’s predictions: (B) number of words in the description, (C) emotional strength, (D) intensity, (E) pleasantness, (F) semantic distance between odors, (G) familiarity, and (H) gender. Each plot shows how the value of a single feature affects the prediction of odor recognition. (I) SHAP waterfall plot illustrating the contribution of features to an individual odor recognition prediction. For this trial, the participant described the odor in 6 words, rated its pleasantness at −3.53, intensity at 7.6, and familiarity at 1.8. The average semantic distance between this odor and the others was 0.97. The participant was female (gender = 0). The horizontal pink and blue arrows represent the contribution of each feature, shifting the model’s mean prediction (*E*[*f*(*x*)] = 0.5) to the specific prediction for this trial (*f*(*x*) = 0.658).

To assess the importance of each feature in predicting odor recognition, we used SHAP values (Figure 4). The three most influential features were the number of words in odor descriptions, odor emotional strength, and odor intensity (Figures 4B–4D). Absence of descriptions (0 words) decreased the model’s output toward recognition, while detailed descriptions (≥4 words) increased it. This contribution plateaued at five words or more. Emotional strength, derived as the absolute value of pleasantness, had a stronger effect than pleasantness itself. Low emotional strength (0) decreased contribution to the model’s prediction toward recognition, while moderate values (1–3) had little effect. In contrast, high emotional strength (>4) contributed more strongly to the model output. Therefore, odors eliciting strong emotional responses contributed more positively to recognition predictions regardless of their valence. According to this SHAP analysis, the number of words and emotional strength exhibited the highest contribution to the model’s predictive performance, suggesting that each captures a distinct and meaningful dimension of odor recognition. Odor intensity followed a similar pattern: low intensities (<3) decreased the prediction toward recognition, moderate intensities (4–7) had minimal effect, and high intensities (≥8) increased it. The effects of odor pleasantness, semantic distance, and odor familiarity were more moderate (Figures 4E–4G). Unpleasant odors (−5 to −2) contributed positively to recognition, while neutral to pleasant odors (−1 to 5) had minimal impact. Regarding semantic distance, low values (<0.4) had minimal impact, moderate distance (0.5–8.0) slightly decreased recognition, and higher semantic distance between odor (>8.0) slightly increased recognition. Odor familiarity had a non-linear effect on recognition, with only extreme ratings influencing the outcome. Low familiarity (0) either decreased or increased recognition, while high familiarity (9–10) was associated with higher recognition. Although the dependence plot shows a slight upward trend for familiarity scores between 1 and 8, the corresponding SHAP values remain close to 0, indicating that this variation had minimal influence on model predictions. Finally, the influence of gender was marginal with SHAP values around 0 (Figure 4H).

SHAP values not only assign weights to features but also enable instance-specific interpretations, allowing the model to generate individual predictions for any given trial. For example, in the trial depicted in Figure 4I, the participant, a woman, rated odor pleasantness at −3.53, intensity at 7.6, familiarity at 1.8, and provided a 6-word description. The semantic distance between this odor and the others was 0.97. Based on these inputs, the model produced a prediction of 0.658 for this trial, compared to the average model output of 0.5 across all trials (*E*[*f*(*x*)]). The most influential feature was the number of words in the description (+0.10 contribution), followed by odor unpleasantness (+0.03) and emotional strength (+0.02). In contrast, gender had no impact (+0.00), while low familiarity slightly decreased the prediction toward recognition (−0.01).

Collectively, these results emphasize that odor recognition was driven primarily by odor emotional characteristics and odor verbal descriptions. These results were consistent with additional statistical analyses (see Figure S4).

### Associative retrieval relies on odor familiarity and semantic distinctiveness

**Figure 5:**
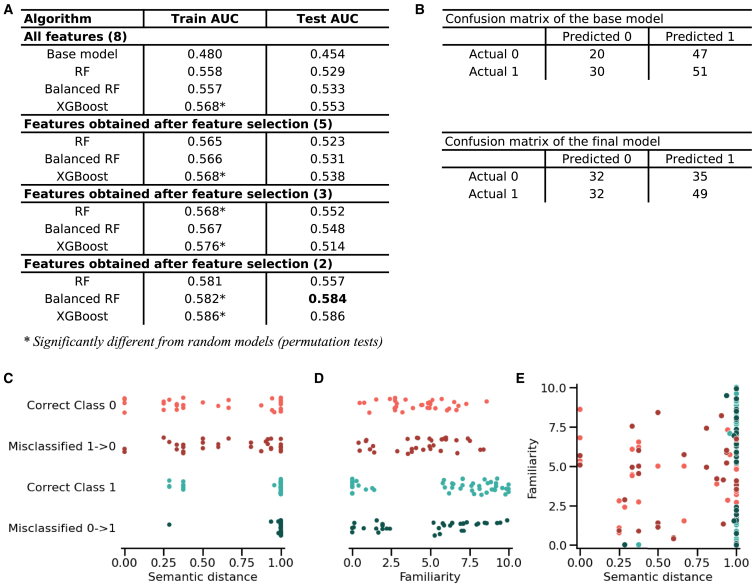
Errors in associative memory reflect feature characteristics (A) AUC scores on training and testing sets across all models. (B) Confusion matrices showing the distribution of actual versus predicted outcomes for the associative component, for both the test set of the base model and the final model. (C and D) Scatterplots showing individual test trials according to: (C) semantic distance between odors and (D) odor familiarity. (E) The feature value ranges of correct predictions misclassified as incorrect is similar to that of trials correctly identified as incorrect, while the distribution feature value ranges of incorrect predictions misclassified as correct is close to that of trials correctly identified as correct. Correct class 0: correctly classified as incorrect associative retrieval (*n* = 32), correct class 1: correctly classified as correct associative retrieval (*n* = 49), misclassified 0–>1: incorrect associative retrieval misclassified as correct associative retrieval (*n* = 35), and misclassified 1–>0: correct associative retrieval misclassified as incorrect associative retrieval (*n* = 32).

The second analysis aimed to predict the associative component of odor-evoked memory among all hit odors. Although the best-performing model was an XGBoost (slightly better AUC performance on both training and test data, Figure 5A), the balanced random forest model presented SHAP values with greater magnitude and a more evenly distributed spread, allowing for a more precise analysis of each variable influence on predictions than the XGBoost (data not shown). Therefore, the balanced random forest model was selected for interpretation. The model consisted of 500 trees, each with a maximum depth of 3 and up to 10 leaf nodes. It used all features for each split (max_features = none), required a minimum of 14 samples per leaf, and ensured a random state of 42 for reproducibility of results. Only two features were retained: semantic distance between odors and familiarity. Permutation tests confirmed that the final model performed significantly better than chance (*p* = 0.04), where the baseline model did not (Figure S3). Examination of the confusion matrices and test set scores further validated the superiority of the final model (Figures 5A and 5B).

To better understand model errors, misclassified trials were analyzed and compared to correctly classified ones (Figures 5C–5E). To visualize these effects, scatterplots were generated to display how feature value ranges differed and overlapped between correct and incorrect classifications. Errors tended to arise in regions of overlapping feature ranges. Correctly predicted retrievals were associated with either moderate (0.25–0.35) or high semantic distance between odors (0.9–1) and familiarity ratings that were either very low (0–2) or high (6–10). In contrast, incorrect retrievals were characterized by semantic distance ranging from 0 to 1 and moderate familiarity (1–8). Misclassified trials reflected the characteristics of their predicted classes. Correct retrievals classified as incorrect resembled incorrect retrievals, showing a higher range of semantic distance and moderate familiarity (1–8). Incorrect retrievals classified as correct mirrored correct retrievals, exhibiting moderate or high semantic distance and lower or higher familiarity. A combined analysis confirmed these patterns (Figure 5E). Errors clustered near their corresponding class distributions, highlighting the overlap between misclassifications and actual classes.

**Figure 6:**
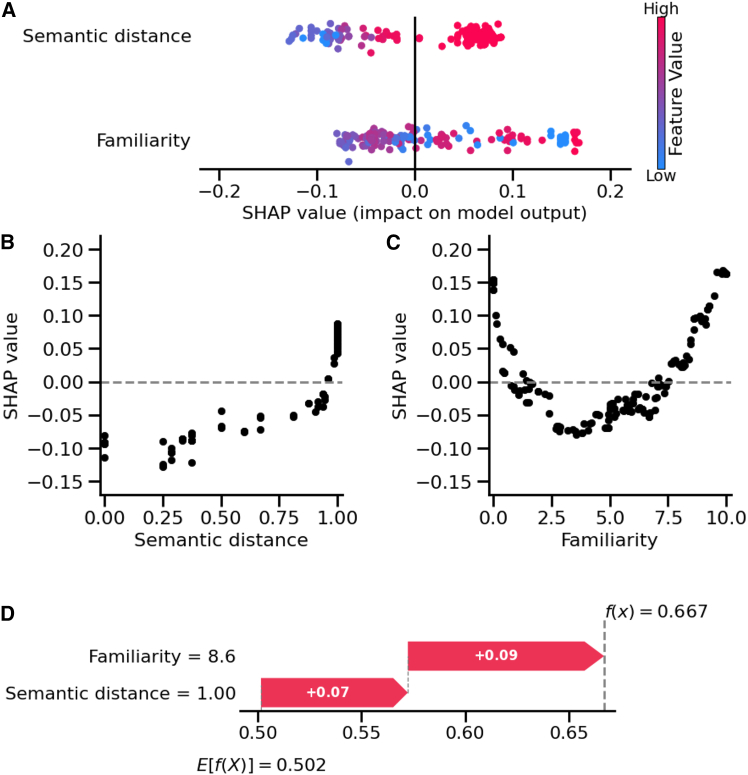
Odor associative memory is primarily driven by odor familiarity and semantic distinctiveness (A) SHAP summary plot showing the contributions of features to associative memory predictions. Each point represents an SHAP value for a single trial, with color indicating the feature’s value (blue for low, pink for high). Positive SHAP values shift the prediction toward correct associative retrieval, while negative values shift the prediction toward incorrect associative retrieval. (B and C) SHAP dependence plots illustrating the effects of (B) semantic distance between odors and (C) odor familiarity on the model’s predictions. The SHAP values, plotted against feature values, show how these features influence the model’s output for predicting associative retrieval, or its absence. (D) SHAP waterfall plot depicting the contribution of features to an individual associative memory prediction. For this trial, the participant rated the odor with a familiarity score of 8.6, and the average semantic distance between this odor and the others was 1. The horizontal pink bars represent the contribution of each feature in shifting the model’s mean prediction (*E*[*f*(*x*)] = 0.502) to the specific prediction for this trial (*f*(*x*) = 0.667).

To evaluate the importance of each feature, we used SHAP values (Figures 6A–6C). The results identified the semantic distance as a slightly more influential predictor of correct associative retrieval than familiarity. Our results showed that a semantic distance <1 reduced the model’s prediction toward associative retrieval while a semantic distance of 1 increased it (Figure 6B). Interestingly, our results indicated that both low familiarity ratings (0–1) and very high familiarity (8–10) were associated with positive SHAP values, indicating higher contributions toward associative retrieval. On the contrary, moderate familiarity scores (2–7) corresponded to negative SHAP values, suggesting reduced contribution toward associative retrieval (Figure 6C).

We also investigated instance-specific interpretations using SHAP values, providing insights into how individual features influenced predictions. For example, in the trial depicted in Figure 6D, the participant rated odor familiarity at 8.6 and the semantic distance between the odor and the others was 1. The model produced a prediction of 0.667 for associative retrieval, compared to the model’s average prediction of 0.502 across all trials (*E*[*f*(*x*)]) (Figure 6D). Odor familiarity contributed to +0.09 and semantic distance further contributed to +0.07.

In summary, these results emphasized that the associative component of odor-evoked episodic memory depends on semantic features, with high semantic distance between odors and familiarity extremes having major contributions. These findings were consistent with additional statistical analyses (see Figure S5).

## Discussion

This study aimed to identify the key predictors of odor-evoked episodic memory by analyzing the influence of perceptual and semantic features of odors, along with participants gender, and by disentangling this complex memory process into two sub-processes: odor recognition and odor associative memory. We trained machine learning models to predict memory performance and used SHAP values to assess features contribution both globally and at the individual trial level.

Odor recognition was best predicted by emotional attributes, and the richness of odor descriptions. Associative retrieval was best predicted by odor familiarity and the semantic uniqueness of odor descriptions. In other words, for an odor to be recognized, it has to be emotional and verbally describable. To go beyond simple recognition and evoke episodic retrieval, an odor had then to be either highly familiar or unfamiliar and semantically distinctive. Overall, this study underlines the importance of both emotional and familiarity idiosyncrasies in shaping olfactory memory and strengthens the crucial role of verbalization in olfactory cognition.

### Emotions enhance odor-evoked episodic memory through odor recognition

Our findings indicated that odor emotional strength and, to a lesser extent, odor pleasantness were key predictors of odor recognition. Odors with strong emotional strength, especially when unpleasant, were more likely to be recognized. Although classical statistical analyses showed a U-shaped relationship with pleasantness, machine learning model revealed that only unpleasant odors were associated with increased recognition, while neutral and pleasant odors had none to low predictive values. These results aligned with previous studies demonstrating enhanced odor recognition for odor with high emotional strength,^11^ especially when unpleasant and intense.^10^ Odor-related emotions are indeed closely linked to their intensity.^11^^,^^42^ It is proposed that odor concentration enhanced pleasantness with pleasant and unpleasant odors being perceived as more pleasant and more unpleasant, respectively.^43^^,^^44^ Accordingly, our results identified odor intensity as another key predictor of odor recognition, with high- and moderate-intensity odors being better recognized than low-intensity ones. Odor intensity and emotional strength may convey overlapping information, such as the probable distance and quantity of an odor source, as well as whether it should be approached or avoided.^42^ Odors associated with strong emotions and perceived as more intense may attract more attention and elicit richer cognitive associations, facilitating their integration into memory and their later recall.^45^^,^^46^^,^^47^ Although these perceptual and affective dimensions are partially correlated, both intensity and pleasantness were retained in the final predictive model after feature selection. This suggests that they capture complementary aspects of the olfactory experience: intensity likely relates to the perceptual magnitude and salience of the stimulus, whereas pleasantness reflects its affective appraisal.

Importantly, our distinctive approach to odor-evoked episodic memory revealed that odor-related emotions selectively enhance odor recognition, without affecting associative retrieval. In other words, odor emotional qualities shape odor-evoked retrieval mainly by increasing the likelihood of odor recognition, sharpening earlier interpretations of the emotion-odor memory link.^48^^,^^49^^,^^50^^,^^51^ Most studies comparing odor-evoked autobiographical memories to those triggered by other modalities (e.g., sounds, images, and tastes) conclude that olfactory autobiographical memories are perceived as more emotional and pleasant.^48^^,^^49^^,^^50^^,^^51^^,^^52^ This strong connection is central to the LOVER model which characterizes olfactory memories as Limbic (greater involvement of the limbic system), Old (retrieved from earlier in life), Vivid (experienced with greater clarity), Emotional (higher emotional intensity), and Rare (less frequently recalled).^2^ Yet, this model has been challenged^4^: while acknowledging the evidence for LOVER, underestimated factors are pointed out, such as semantic processing and differences between direct and indirect retrieval, and caution is required against assuming that olfactory memories are inherently emotional. Instead, it is argued that odor’s emotional properties may bias subsequent appraisals of the memory. Supporting this, a study shows that when participants recalled odor-associated autobiographical memories without a sensory cue, the memories are no more emotional or older than those triggered by other modalities.^53^ Specific odor attributes may therefore modulate the emotionality of odor-evoked memories. For example, childhood-related odors have been found to elicit more emotionally intense recollections than childhood images or non-childhood odors and images, likely because these odors carry strong personal significance.^52^ Similarly, it is reported that only a subset of odors evoked emotional autobiographical memories,^54^ suggesting that particular, yet unmeasured, perceptual qualities enable certain odors to evoke especially vivid and emotional memories.

In brief, by disentangling the two sub-processes of episodic memory (*i.e*., odor recognition and associative retrieval), our study demonstrates that odor emotional qualities boost odor-evoked episodic retrieval by enhancing odor recognition. This provides the first direct evidence that the emotion-memory link for odors operates primarily at the cue-recognition stage rather than through associative binding. It contributes to growing evidence for the tight coupling between olfaction and emotion.^55^^,^^56^

### Nonlinear influence of familiarity: How novel and highly familiar odors enable episodic memory through associative retrieval

Our results showed that odor familiarity was a key predictor of odor associative retrieval. When an odor was correctly recognized, both extremes of the familiarity continuum enhanced retrieval: completely unfamiliar or highly familiar odors increased the likelihood of remembering the associated context, whereas moderately familiar odors tended to limit it. This extends findings reporting that familiar odors evoked more autobiographical memories.^48^ Indeed, in contrast to our controlled yet ecologically valid design, autobiographical paradigms cannot test unfamiliar odors which, by definition, have never been experienced and, therefore, cannot trigger a personal memory.

The Misfit Theory of Spontaneous Conscious Odor Perception offers a relevant framework for understanding why unfamiliar odors increased associative binding.^57^ It proposes that we become consciously aware of an odor primarily when it mismatches expectations, and that olfactory memory functions less to remember or identify odors and more to flag novelty. Once familiar, the odor usually fades from conscious awareness, and its first association tends to persist, because later encounters do not overwrite it. This persistence is clear in classic odor-picture associations studies, where the initial pairing is retained better than subsequent ones,^58^ and leaves a particularly strong hippocampal memory trace.^59^ In our experiment, the first encounter with a novel odor may have benefited from this privileged initial encoding.

In contrast, the robust associative retrieval observed for highly familiar odors likely draws on preexisting semantic and autobiographical knowledge. Given that highly familiar odors are more likely to be named,^14^^,^^15^^,^^16^ their association with the encoding context may not have been based on their olfactory perceptual features alone, but also on their verbal or conceptual representation. Growing evidence shows that semantic and episodic memories are closely interconnected systems.^60^ Semantic knowledge is thought to provide a structured framework that influences episodic memories, facilitating their organization, accessibility, and enrichment. During encoding, it enables complex events to be segmented and memorized based on preexisting schemas^61^^,^^62^ and, during retrieval, it helps structure and complete memories, notably by activating semantic networks that interact with episodic ones.^63^^,^^64^ Neuroimaging studies support these distinctions: familiar odors engage regions involved in semantic association and episodic retrieval (left prefrontal cortex, inferior frontal gyrus, parieto-occipital regions, and medial temporal lobes), whereas unfamiliar odors recruit regions linked to novelty detection, with increased activation in the right insula.^65^^,^^66^

In sum, odor familiarity exerts a pronounced, non-linear influence on odor-evoked episodic memory: both novel and highly familiar odors facilitate associative retrieval, likely through different cognitive and neural processes. Treating familiarity as a continuous bimodal variable, rather than a binary one, will be essential for future research on olfactory cognition.

### Verbal odor descriptions support odor-evoked episodic memory: Richness enhances recognition, distinctiveness improves associative retrieval

A wealth of research highlights human limitations in naming, categorizing, or describing odors.^15^^,^^55^^,^^67^^,^^68^ Yet in our study, participants still produced verbal descriptions for a majority of odors, despite odors being only moderately familiar and considerably difficult to identify. Two semantic features extracted from those odor descriptions, richness (number of words) and distinctiveness (semantic distance from a participant’s other descriptions), had dissociable effects on the sub-processes of odor-evoked memory. Richer descriptions improved odor recognition, whereas more distinctive descriptions enhanced associative retrieval.

For odor recognition, providing no description was associated with slightly lower performance, while longer descriptions predicted higher recognition rates. Although semantic knowledge of odors, often assessed by their correct identification, is typically linked to greater odor familiarity,^14^^,^^15^^,^^16^ familiarity cannot be directly inferred from the numbers of words used in a description. A brief description can reflect either precise labeling (e.g., “*eucalyptus*”) or scant information (e.g., “*unknown*”), while an extended description might signal rich semantic knowledge (e.g., “*Vicks cream applied under the nose when it’s blocked, eucalyptus*”), or uncertainty (*e.g*., “*It reminds me of something, maybe sweet almond but not sure*”). Participants also inserted emotional reactions (e.g., “*unpleasant*”) or surprise (e.g., “*weird*”) which, while not providing semantic content, may still anchor the odor in memory and enhance recognition. Prior studies reported mixed results on the role of semantic information in olfactory memory. Some show weak links between semantic knowledge and odor recognition,^12^^,^^21^^,^^22^ while others suggest that the ability to identify familiar odors or assign predetermined labels to unfamiliar odors facilitates recognition.^14^^,^^17^^,^^18^^,^^19^^,^^20^ Interestingly, incorrect but consistent description between encoding and retrieval phases also helps odor recognition, suggesting that “unconventional” or “non-normative” descriptions may still reflect meaningful olfactory knowledge.^18^^,^^69^^,^^70^ Regarding odor-evoked memory, correctly named odors are reported to be more likely, but not essential, to trigger autobiographical memories.^48^ In our study, as we collected odor descriptions after retrieval to avoid interference at encoding, and used complex unfamiliar stimuli (e.g., dihydromyrcenol and methyl octine carbonate), hard to label correctly, we could not test description consistency or correctness. Our results nonetheless indicate that any verbal description increases the odds of later recognizing the odor.

To address the independence between the two first factors explaining odor recognition, we additionally investigated whether emotionally potent odors might lead to richer descriptions. No significant correlation between emotional strength and the number of words was found (data not shown), and generalized linear mixed models showed that the number of words contributed to recognition independently of emotional strength (data not shown). This suggests that verbal descriptions provide complementary information to emotional salience, rather than merely reflecting it.

Odor associative retrieval was best predicted by the semantic distance between odor descriptions. Odors described with unique terms (i.e., sharing no words with a participant’s other descriptions) were more likely to be recalled with the correct context, probably because distinct descriptions reduce conceptual overlap. In contrast, shared descriptive elements between odor descriptions may blur conceptual representations, increasing the risk of contextual confusion during encoding. This aligns with neural evidence that perceptually similar odors elicit overlapping patterns in the piriform cortex, while discrimination learning leads to more distinct representations.^71^^,^^72^ Taken together, our results show that verbalizations, however imprecise, benefit odor-evoked memory. Odor recognition relies on descriptive richness, whereas associative retrieval is enhanced by semantic uniqueness. Far from being a “muted sense,”^73^ olfaction gains structure from language, even when labels are approximate. Continuing to develop tools for analyzing olfactory language and misnaming^74^^,^^75^^,^^76^ will therefore be crucial for better characterizing the cognitive mechanisms that link odors, language, and memories.

In summary, by analyzing odor recognition and associative retrieval separately, our study provides new insights into how perceptual, emotional, and semantic factors differentially shape odor-evoked episodic memory. Odor recognition is primarily driven by odor emotional strength, mainly when unpleasant, and by the richness of its description. Associative retrieval is influenced by odor familiarity in a U-shaped way, as both novel and highly familiar odors increase episodic association retrieval, likely through distinct mechanisms: privileged initial encoding for novel odors and semantic scaffolding for familiar ones. In addition, semantic distinctiveness among odor descriptions facilitates associative retrieval, likely by reducing conceptual overlap.

Overall, odor-evoked episodic memory depends not only on odor emotional strength but also on how an odor is conceptualized and our acquaintance with it. Odors lacking emotional salience or poorly verbalized tend to be forgotten. Conversely, emotionally potent odors trigger memory processes ranging from recognition alone to the full episodic retrieval, depending on the richness and uniqueness of their verbal descriptions. To be truly “Proustian,” an odor should be both emotional and semantically distinctive.

Our findings place verbalization, long considered the weak link of olfaction, at the center of olfactory cognition research, and they illustrate the value of machine-learning approaches despite the data’s inherent variability.

### Limitations of the study

This study has several limitations. First, gender and pleasantness showed partial discrepancies between model predictions and statistical analyses. Gender contributed only marginally to model accuracy, yet statistical analyses revealed that women outperformed men in odor recognition, consistent with literature.^77^^,^^78^^,^^79^ Pleasantness also differs, as our model revealed that only unpleasant odors increased recognition, while statistical analyses showed a U-shaped relationship between pleasantness and recognition. Including highly pleasant odors in a future study would test whether positive valence also improves odor recognition. Second, model performance was limited for predicting incorrect odor recognition, and associative memory. This may reflect insufficient data for the model to learn consistent patterns, as sparse examples reduce its ability to generalize. Larger datasets may improve predictive performance. Third, our odor set may not have spanned the olfactory perceptual space enough for robust classification. Adding more complex odors and getting data from a more culturally and genetically diverse population should expand the space captured and reduce class overlap.

Finally, our feature set may lack olfactory dimensions. Integrating richer verbal descriptions and using recent natural language processing tools could better capture the structure and content of olfactory language,^74^^,^^75^^,^^76^ as our results reinforce the importance of odor language in odor-evoked memory.

## Resource availability

### Lead contact

Further information and requests for resources and information should be directed to and will be fulfilled by the lead contact, Anne-Lise Saive (alsaive@institutlyfe.com).

### Materials availability

This study did not generate new unique reagents.

### Data and code availability

• The behavioral dataset generated for this study has been deposited at OSF and is publicly available as of the date of publication. See key resources table for the repository link.
• All original code has been deposited at GitHub and is publicly available as of the date of publication. See key resources table for the repository link.
• Other items: Any additional information required to reanalyze the data reported in this paper are available from the lead contact upon request.

## Acknowledgments

We thank Aurélie Coubart and David Morizet for their discussions and insights throughout the project. Juliette Greco-Vuilloud was founded by L’Oréal Research & Innovation.

## Author contributions

Conceived and designed the experiments, J.G.-V., J.P., and A.-L.S.; collected the data, P.R., J.P., and A.-L.S.; analyzed the data, J.G.-V.; wrote and revised the article, J.G.-V., J.P., and A.-L.S.

## Declaration of interests

The authors declare no competing interests.

## STAR★Methods

### Key resources table

**Table:** 

REAGENT or RESOURCE	SOURCE	IDENTIFIER
**Deposited data**		

Dataset	This paper	https://osf.io/m5236/

**Experimental models: Organisms/strains**		

Human adults	Recruited in France	

**Software and algorithms**		

Python (v3.11)	Python Software Foundation	https://www.python.org/
NLTK	Bird et al.^80^	https://www.nltk.org/
spaCy	Explosion AI	https://spacy.io/
Pandas	McKinney et al.^81^	https://pandas.pydata.org/
scikit-learn	Pedregosa et al.^82^	https://scikit-learn.org/
imbalanced-learn	Lemaître et al.^83^	https://imbalanced-learn.org/
xgboost	Chen and Guestrin^84^	https://xgboost.readthedocs.io/
scikit-optimize	Head et al. 2018^85^	https://scikit-optimize.github.io/
SHAP	Lundberg and Lee^86^	https://github.com/slundberg/shap/
R software (v4.3)	R Core Team	https://www.r-project.org/
LabView (≥v8.6)	National Instruments	https://www.ni.com/
The code is publicly available at GitHub	This paper	https://github.com/jltt-grc/memorable_scents/

**Other**		

20-channel computerized olfactometer	Sezille et al.^87^	N/A

### Experimental model and study participant details

#### Human participants

This study was conducted by pooling data from four independent studies, three published^11^^,^^16^^,^^40^ and one unpublished, all following the same experimental paradigm based on Saive et al. 2013.^88^ As one study specifically recruited high dream recallers^40^ while the others did not, we statistically verified that participants’ performance did not differ significantly across studies, indicating that the datasets were broadly comparable in terms of performance and experimental conditions (data not shown).

A total of 106 French participants (65 women, 41 men), aged 22.0 ± 2.3 years (mean ± SD), were included from the four studies (Table S2). Differences between men and women were assessed: women showed a higher probability of success in the odor recognition task (Figure S4), while group-related effects were minimal in the machine-learning analyses and did not influence model performance (Figure 4).

All studies were conducted in compliance with French regulations governing biomedical research on healthy volunteers and adhered to the principles of the Declaration of Helsinki. Each study was approved by the local institutional review board (Comité d'Éthique du CPP Sud-Est IV, approval IDs: 2010-A-01529-30 and 2015-A01595-44). All participants provided written informed consent and received financial compensation for their participation.

### Method details

#### Odorants and delivery

Each individual study involved 18 odorants. These odorants included essential oils, monomolecular chemical compounds or mixtures, selected for their distinctiveness as well as their relatively moderate intensity, familiarity, and identifiability (Table S3). In each study, odorants were divided into two sets (target and distractor) of nine odorants each.

Odorants were administered directly to the entrance of participants' nostrils using a 20-channel computerized olfactometer that synchronized odorant diffusion with the participants' breathing.^87^ The respiratory signal, acquired via a nasal cannula, was used to trigger odor stimulation using an airflow sensor. The flow rate was set at 3 L/min and each stimulation lasted 4 s.

#### Episode creation

In each study, three multidimensional episodes were designed, each integrating three odors (What), associated with specific locations (Where), represented by orange circles, within a given visual context (Which) consisting of a landscape picture, thereby satisfying the simplified content-definition of episodic memory.^89^^,^^90^ To limit semantic processing, the odors, spatial locations, and visual context were arbitrarily linked, and their associations varied for each participant. Episodes were presented using LabView software (version 8.6 or higher). Participants interacted with the software via a trackball (Kensington, USA). When participants clicked on a circle, an odor was delivered at the beginning of the next exhalation, ensuring its perception during the subsequent inhalation.

#### Experimental paradigm

All four studies used the same experimental paradigm designed to investigate episodic memory in a controlled and ecologically valid manner, closely simulating real-life episodes.^3^^,^^88^ The detailed experimental procedures for each study are thoroughly described in the original publications.^11^^,^^16^^,^^40^ A summary of the common paradigm used across all four studies is provided here for clarity (Figure 1). The paradigm consisted of two phases: an encoding phase over three days and a recall phase on the fourth day.

##### Encoding

During encoding, participants freely explored one episode per day for 7 min over a total of three days. They were encouraged to explore the landscapes, the positions of the circles, and the associated odors in detail by clicking on the circles. However, no explicit instructions to memorize the content were provided. The order of the episodes was randomized across participants.

##### Retrieval

Episode retrieval took place on day 4. Each trial began with an odor recognition task in which participants were presented with either one of the nine target odors (previously encountered during the encoding phase) or one of the nine distractor odors (new odors). They were instructed to respond “Yes” if they recognized the odor as having been previously presented or “No” if they did not. If participants responded “Yes”, the trial proceeded to the associative part of the episodic retrieval task, in which they were asked to first select a context from the three landscape pictures, then a location from the nine circles.

##### Odor ratings

At the end of the experiment, participants rated the odors in terms of pleasantness, intensity, and familiarity using bounded, unmarked linear scales. The pleasantness scale ranged from “*extremely unpleasant*” to “*extremely pleasant*”, with a “*neutral*” midpoint. The intensity scale ranged from “*extremely weak*” to “*extremely strong*” and the familiarity scale ranged from “u*nknown/not familiar*” to “*very well-known/extremely familiar*”. An additional dimension, emotional strength, was calculated as the absolute value of pleasantness, reflecting the emotional intensity of the odor regardless of valence.^42^ The mean perceptual ratings and the correlations between the sensory features are represented in Figure S6.

##### Odor descriptions

Participants were also asked to provide free descriptions of the odors, whenever possible. These descriptions included any terms the participants used to describe the odors, (e.g., odor sources, metaphors, feelings, emotions) in any format they preferred (e.g., sentence, list of words). Examples of such descriptions include: “*Medication used during childhood. Blue/purple tube, more or less white translucent paste. Brand name 'Vicks'*”, “*Intense scent, not really pleasant*”, “*Cheese*”. These examples are presented in English for clarity, but all descriptions were originally written and analyzed in French and subsequently preprocessed to ensure uniformity and facilitate analysis. Preprocessing involved multiple steps, starting with the removal of punctuation, special characters, and common stop words such as articles and conjunctions. Additional domain-specific stop words, including “odor,” “odors,” “smells,” and “smelled,” were also removed, as they did not bring semantic meaning to the descriptions.

Following this, all text was normalized by converting words to lowercase to maintain consistency. The descriptions were then tokenized, dividing the text into individual words or tokens, which allowed for more granular processing. Each token was further lemmatized, reducing it to its base form, or lemma (e.g., “sweetness” was reduced to “sweet” and “freshness” to “fresh”), to simplify analysis and reduce lexical variability. These preprocessing steps ensured that only meaningful content was retained, enabling a systematic evaluation of the descriptions. All preprocessing steps were implemented using NLTK,^80^ spaCy, and Pandas^81^ libraries in Python.

### Quantification and statistical analysis

The primary aim of this study was to predict memory performance, specifically the accuracy of odor recognition and the accuracy of odor associative retrieval, using a range of features derived from experimental data and participant responses**.** These predictive features were hypothesized to influence both memory performance and were incorporated into the prediction models. The distributions of all the variables are presented in Figure S1 and described in detail below.

#### Memory performance

Two binary outcome variables were predicted: (1) the accuracy of odor recognition and, (2) the accuracy of odor associative retrieval for correctly recognized odors.

Odor recognition accuracy assessed whether participants correctly remembered a previously encountered odor (target). A *hit* referred to a correctly recognized target odor, while a *miss* referred to a target odor incorrectly rejected as new. Analyses focused exclusively on target odors (*n* = 949 trials; Figure S1A), with hits coded as 1 (*n* = 736) and misses as 0 (*n* = 213). False alarms (i.e., incorrect recognition of a distractor odor) and correct rejections (i.e., accurate rejection of a distractor odor) were excluded, as the primary aim was to model the accuracy of odor recognition for previously encountered odors, not responses to novel odors.

Odor associative retrieval was evaluated only for odors that were correctly recognized (hits). Participants were asked to retrieve contextual details about the odor, consisting of the visual context (landscape image) and spatial location (circle position). The associative retrieval was considered correct if participants selected at least the correct context. Analyses were restricted to hits (*n* = 736 trials; Figure S1I). Correct retrievals were coded as 1 (*n* = 405 trials), and incorrect retrievals were coded as 0 (*n* = 331 trials).

#### Predictive features

##### Gender variable

Participants' gender was encoded as a binary variable, with 0 corresponding to women (W) and 1 to men (M).

##### Perceptual variables

Participants’ subjective sensory evaluations were used as predictive features to assess their influence on memory performance. As the original rating scales were ungraduated, the sensory evaluations were transformed *post hoc* into numerical scores. Pleasantness ratings were transformed into scores ranging from −5 to 5, with 0 indicating neutrality. Emotional strength was transformed into scores ranging from 0 to 5. Intensity and familiarity ratings were also transformed into scores ranging from 0 to 10.

##### Semantic variable

One variable was created based on the processed descriptions when provided. The variable Number of Words corresponded to the total number of words in each description, computed after preprocessing, to assess the richness of odor descriptions.

##### Perceptual distance

To *determine* whether perceptual distinctiveness influenced memory performance, we computed perceptual distances between odors based on participants’ ratings of pleasantness, intensity, and familiarity. Emotional strength was excluded, as it is a derived measure of pleasantness. Each odor was represented as a point within a three-dimensional perceptual space specific to each participant and memory task. For odor recognition, perceptual distances were computed for all target odors (Figure 2A). For odor associative retrieval, distances were computed only for correctly recognized odors (hits; Figure 2B). Euclidean distances were used to calculate the pairwise distance between odors, and these values were averaged to yield a mean perceptual distance for each odor within each participant’s perceptual space. The formula for the Euclidean distance between two odors, A and B, is as follows:

$${\text{ED}}_{\left(A,B\right)}=\sqrt{{\left(P_{A}-P_{B}\right)}^{2}+{\left(F_{A}-F_{B}\right)}^{2}+{\left(I_{A}-I_{B}\right)}^{2}}$$

where *P*, *F* and *I* represent ratings for pleasantness, familiarity, and intensity. A perceptual distance close to zero indicates that an odor is perceptually similar to the others within the same participant’s ratings, whereas a higher perceptual distance reflects greater distinctiveness relative to the other odors rated by that participant.

##### Semantic distance

To explore the relationship between semantic variability and memory performance, we computed semantic distances between odor descriptions based on the Jaccard distance.^91^ This metric quantifies the dissimilarity between two sets of words by comparing their overlap relative to their union. Specifically, the Jaccard distance is defined as:

$$JD_{\left(A,B\right)}=1-\frac{\left|A\cap B\right|}{\left|A\cup B\right|}$$

where *A* and *B* represent sets of lemmas derived from odor descriptions. The Jaccard distance ranges from 0 (identical sets) to 1 (completely disjoint sets). Two empty sets, i.e., with no description, were considered equal, and their Jaccard distance was therefore set to 0.

This approach was chosen over more complex measures, such as cosine similarity or word embeddings, as those methods require complete textual data, which was not consistently available. Thus, the Jaccard distance allowed us to accommodate missing descriptions, ensuring all trials could be included in the machine learning pipeline. As with the perceptual distance, the semantic distance was computed separately for each memory task. For each participant, Jaccard distances were computed between pairwise odors, and these distances were averaged to yield a mean semantic distance for each odor within that participant’s set of descriptions.

#### Data exclusion

Following data inspection, out of a total of 1,908 trials (106 participants ∗ 18 odors), ten trials from nine participants were excluded because the participants did not evaluate pleasantness, intensity, or familiarity, resulting in missing values. This exclusion left 1,898 trials available for the final analysis, comprising 949 target odor trials and 949 distractor odor trials. Since we intended to use outlier-robust machine learning models, no further data were removed.

#### Machine learning

##### Model selection

Random Forest and XGBoost classifiers were selected for predicting both odor recognition and odor associative retrieval due to their robustness to outliers and ability to handle both linear and non-linear relationships between variables, including potential interactions. Specifically, they were used to predict (1) the correct recognition of target odors and (2) the correct associative retrieval for hit odors.

Given the class imbalance observed in both memory tasks (736 hits vs. 213 misses for odor recognition and 405 correct vs. 331 incorrect retrievals for associative retrieval) we implemented different strategies to mitigate its impact. For odor recognition, where class imbalance was particularly pronounced, we tested three approaches: Balanced Random Forests, Random Forests with Synthetic Minority Over-sampling Technique for Nominal and Continuous variables (SMOTE-NC), and the scale_pos_weight parameter in XGBoost. For odor associative retrieval, although the class imbalance was more moderate, we tested standard Random Forests, Balanced Random Forests, and adjusted scale_pos_weight in XGBoost to ensure balanced learning. Balanced Random Forests adjusted class weights to compensate for the imbalance (see BalancedRandomForestClassifier). SMOTE-NC generated synthetic samples to balance classes while accounting for continuous and categorical variables (see SMOTE-NC). Random Forest and Balanced Random Forests were implemented using the scikit-learn library,^82^ SMOTE-NC was implemented using the imbalanced-learn library,^83^ and XGBoost was implemented using the xgboost library.^84^

##### Train/test splitting

Before training, the data was divided into training and testing sets to respectively optimize model parameters and evaluate generalization to unseen data. The StratifiedGroupKFold method with five splits from the scikit-learn library was used for the split (see StratifiedGroupKFold). Stratification preserved class distributions in each fold, while grouping by participants ensured that data from the same participant did not appear in both training and testing sets. This reduced the risk of overfitting and improved model generalization. For the odor recognition component, the training set included 581 hits and 170 misses, with the test set contained 155 hits and 43 misses. For the odor associative component, the training set included 324 correct and 264 incorrect retrievals, with the test set containing 81 correct and 67 incorrect retrievals.

##### Model training

For both predictions, a baseline model (i.e., unoptimized and unbalanced) Random Forest Classifier was first created as a benchmark for comparison with more complex models. This baseline included all features in the dataset: gender, pleasantness, emotional strength, intensity, familiarity, perceptual distance, number of words, and semantic distance.

Model optimization was improved by hyperparameter tuning using BayesSearchCV from the scikit-optimize library^85^ (see BayesSearchCV) and feature selection. The hyperparameter tuning for the Random Forests included: the number of trees (500–2000), the maximum tree depth (3–50) and maximum leaf nodes (10–300), the minimum samples per leaf (1–20), the minimum samples to split nodes (2–20), and the number of features considered for splits (“sqrt,” “log2,” or none). The hyperparameter tuning for the XGBoosts included: the number of trees (500–2000), the maximum tree depth (3–50) and maximum leaf nodes (10–300), the minimum samples per leaf (1–20), the minimum samples to split nodes (2–20), and the number of features considered for splits (“sqrt,” “log2,” or none). The hyperparameter tuning for XGBoost included: the number of trees (500–2000), the maximum tree depth (3–50), the maximum number of leaves (10–300), the minimum child weight (1–20), the gamma parameter (0–10), the subsample ratio (0.5–1.0), the proportion of features used for each tree (0.25, 0.5, or 1), and the number of parallel trees (1–10).

The best model was identified based on its ability to distinguish between classes, measured by the receiver operating characteristic-area under the curve (ROC-AUC).

##### Feature selection

Feature selection was assessed using SHapley Additive exPlanations (SHAP) values from the SHAP library^92^ on the training sets to identify variables most predictive of performance.^93^^,^^94^ SHAP values assign contributions to each feature by modeling predictions as a cooperative game, where each feature (or “player”) contributes to the prediction (or “payoff”). These contributions are distributed fairly based on each feature marginal contribution to the outcome. SHAP values were analyzed in all trained models, and features classified as less important in most models were removed. Positive SHAP values indicate that a feature shifted the model’s prediction toward class 1 (correct memory performance), while negative SHAP values shifted it toward class 0 (incorrect performance). SHAP values close to zero suggest that a feature has minimal influence on the prediction.

##### Permutation testing for model comparison

To ensure that model performance was not due to random chance, permutation testing (scikit-learn library) was applied to the training set.^95^ The target variable was shuffled 100 times while preserving the feature distributions. Models were retrained for each permutation, and permutation performance metrics were compared to the actual model performance. *p*-values were calculated as the proportion of permuted models performing as well as or better than the actual model. A significant threshold of α = 0.05 was used, with *p*-values below 0.05 being considered statistically significant.

##### Model evaluation

Model performance was evaluated using a confusion matrix and ROC-AUC score. A confusion matrix is a table that summarizes the model’s classification performance by displaying the number of true positives (correctly predicted positives), true negatives (correctly predicted negatives), false positives (incorrectly predicted positives), and false negatives (incorrectly predicted negatives). It provides a detailed breakdown of prediction errors, helping to assess the model’s strengths and weaknesses. ROC-AUC measured the model’s ability to distinguish between classes, with values close to 1 indicating excellent performance and values near 0.5 indicating random performance.

##### Error analysis

To explore patterns in model errors, we visualized error distributions for each feature, comparing them to distributions of correctly classified trials. For cases involving more than two features, t-SNE (t-distributed Stochastic Neighbor Embedding) was applied to reduce dimensionality and project data into two-dimensional space.^96^ The scikit-learn library was used, with perplexity set at 30.

##### Interpretability

To interpret the models’ predictions, we relied on SHAP values, which provide instance-specific insights into how individual features influenced each prediction. SHAP values were visualized for selected models to identify feature contributions across prediction outcomes.

#### Additional statistics

##### Memory variables

To validate the actual impact of the final features selected in each machine learning model on odor recognition and odor associative memory, we complemented our study with statistical analyses.

We individually tested each predictor variable to assess its independent effect on odor recognition and odor associative memory, while accounting for the hierarchical structure of the data. Generalized Linear Mixed Models (GLMMs) were implemented using the glmmTMB package in R. For the odor recognition task, the GLMMs included participants, study, and odor identity as random effects, accounting for repeated measures within individuals, differences across the four pooled studies, and the fact that the same odors were smelled by multiple participants. For the odor associative memory task, only participants were included as a random effect. These random effect structures were selected because they minimized the Akaike Information Criterion (AIC), following best practices in model selection.^97^

This approach ensured that the predictors were both statistically robust and relevant to the memory outcomes. By integrating machine learning and statistical modeling, the analyses provide complementary insights into the factors influencing odor-evoked episodic memory performance.

##### Perceptual variables

To explore the relationships between the sensory features, we calculated the mean ratings of pleasantness, intensity, and familiarity across the 21 odors. Given that pleasantness was assessed on a bipolar scale, the data were processed in two ways: emotional strength, defined as the absolute value of the pleasantness rating, and valence, defined by the sign of the rating. Odors rated between −5 and 0 were categorized as unpleasant, while those rated between 0 and +5 were categorized as pleasant, with averages calculated separately. Relationships between these perceptual variables were analyzed using Pearson correlations in R. To control for multiple comparisons, Holm’s correction was applied to the *p*-values.

For the statistics, a *p*-value of <0.05 was considered statistically significant. In the figures, ns indicates non-significant differences, ∗ indicates *p* < 0.05, and ∗∗ indicates *p* < 0.01.
