# Supplementary material for: What makes a scent trigger a memory? A cognitive decomposition of odor-evoked retrieval
Source: iScience. 2025 Dec 17;29(1):114467. doi: 10.1016/j.isci.2025.114467 (PMC12808892; doi:10.1016/j.isci.2025.114467)
Supplement: Document S1. Figures S1–S6 and Tables S1–S3 [file mmc1.pdf]

iScience, Volume *29*

Supplemental information

What makes a scent trigger a memory?

A cognitive decomposition

# of odor-evoked retrieval

Juliette Greco-Vuilloud, Perrine Ruby, Jane Plailly, and Anne-Lise Saive

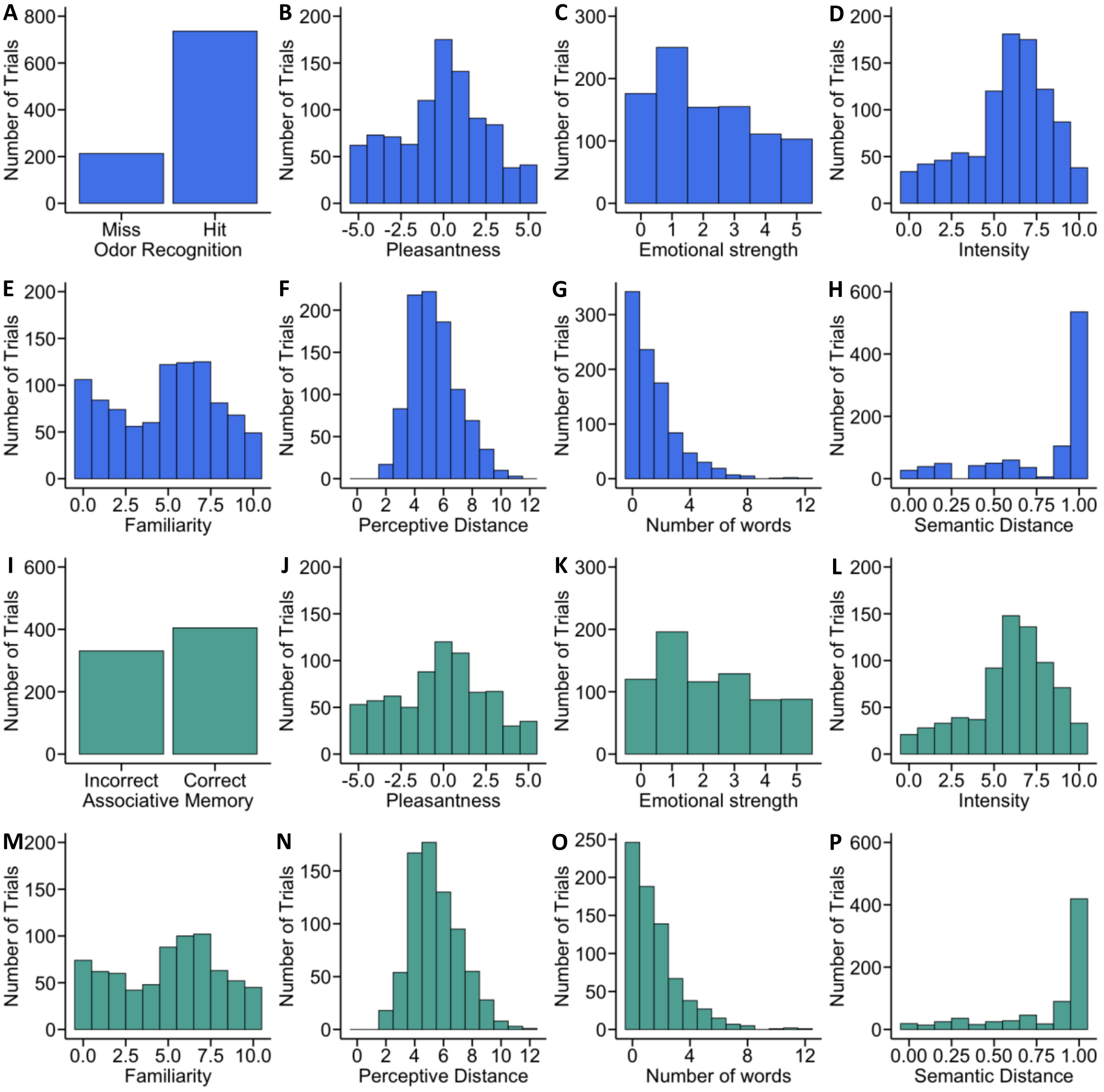

Figure S1. Distributions of variables used for predicting odor recognition and associative memory performance.

(A-H) In blue, distributions of variables used in the odor recognition prediction task: (A) Binary outcome variable for odor recognition prediction, (B-E) Distributions of sensory features (pleasantness, emotional strength, intensity, familiarity) for all target odors, (F) Distribution of the perceptive distance between target odors, (G) Distribution of the number of words used to describe the target odors, (H) Distribution of the semantic distance between target odors. (I-P) In green, distributions of variables used in the associative memory prediction task: (I) Binary outcome variable for associative memory prediction, (J-M) Distributions of sensory features (pleasantness, emotional strength, intensity, familiarity) for hit odors, (N) Distribution of the perceptive distance between hit odors, (O) Distribution of the number of words used to describe the hit odors and (P) Distribution of the semantic distance between the hit odors.

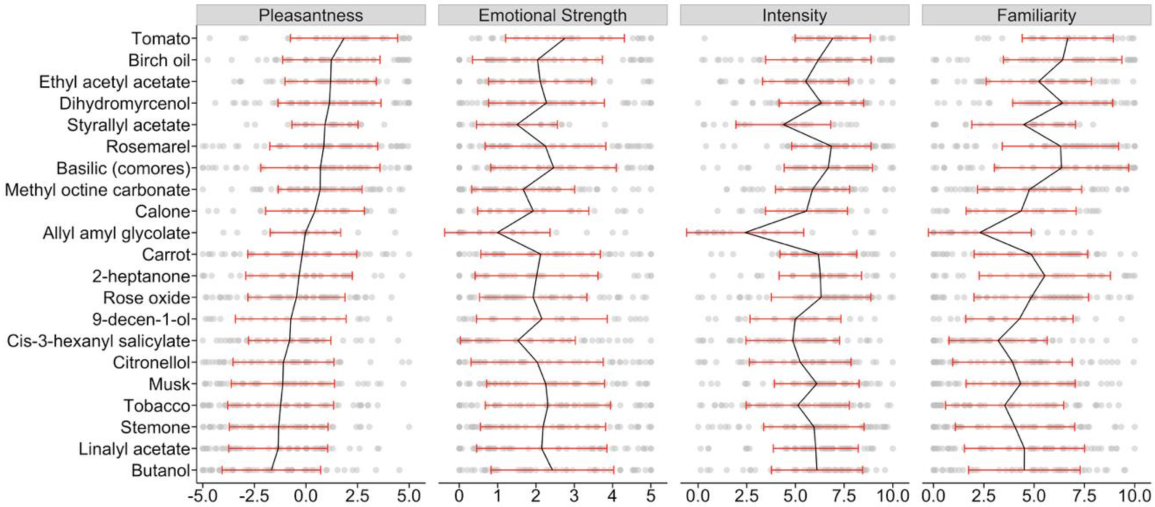

Figure S2. High variability in perceptual ratings across target odors.
Grey dots represent individual ratings from 106 participants for each odor on four dimensions. The black line indicates the average rating for each odor, and the red bars represent the standard deviation.

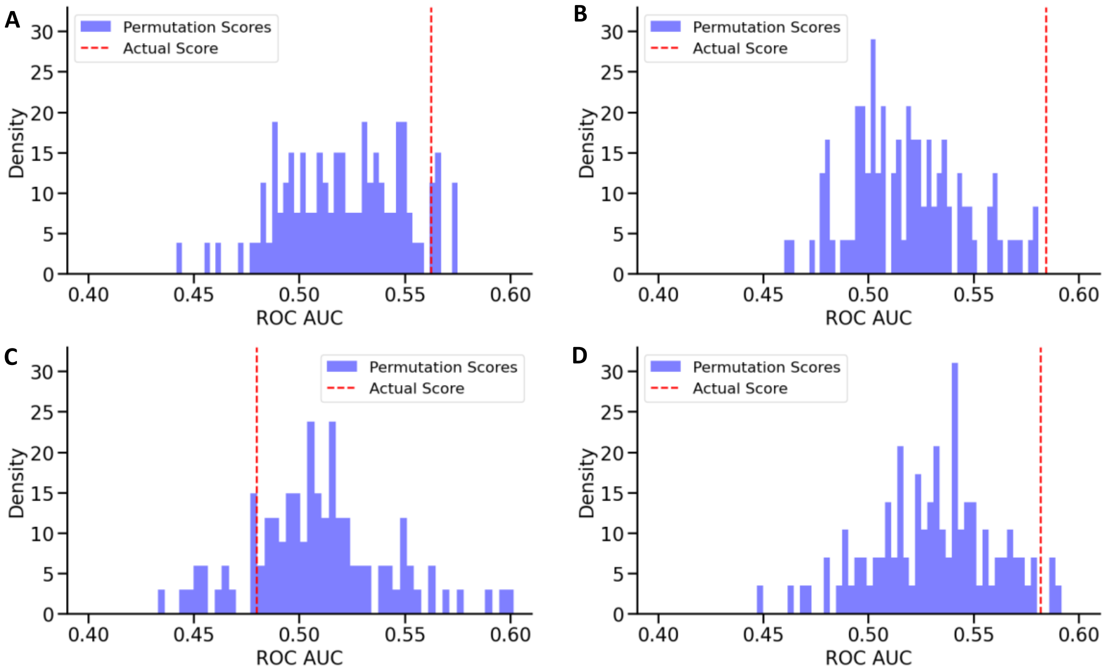

Figure S3. Optimized models outperform baseline classifiers in odor memory prediction. Results of the permutation test performed on the training set for the (A) odor recognition component base model, (B) odor recognition component final model, (C) associative memory component base model, and (D) associative memory component final model. The red dashed line represents the actual ROC-AUC score on the training set, while the blue histogram shows the distribution of ROC-AUC scores for randomly permuted labels.

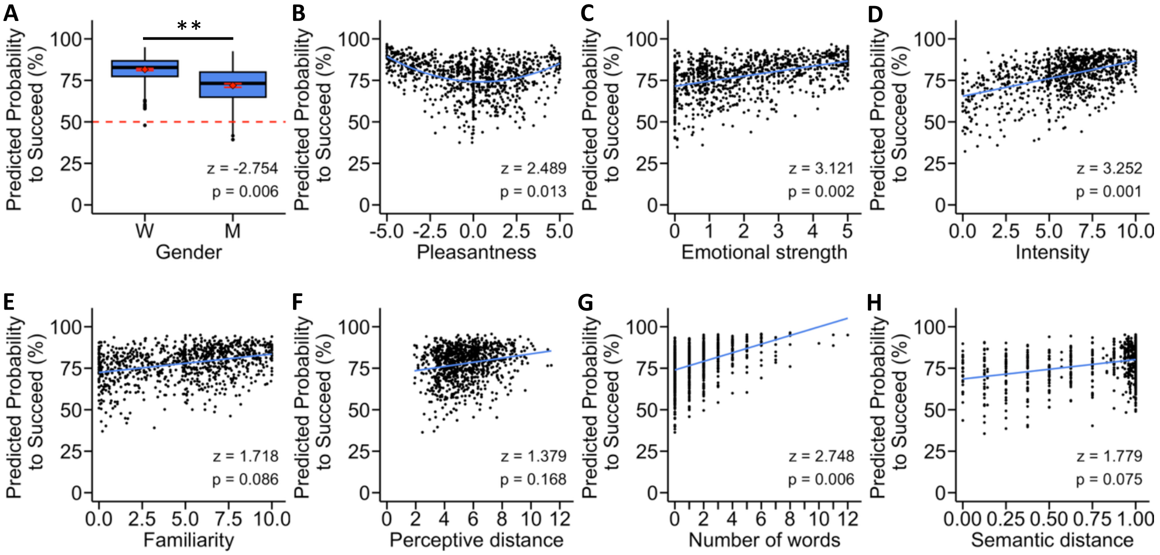

Figure S4. Individual predictors of odor recognition replicate machine learning findings. (A) Impact of gender (binary: women vs. men) on the predicted probability to correctly recognize an odor. The red dashed line corresponds to the chance level. The distribution of the predicted probabilites is displayed using boxplots in black. Estimated means are represented by a red dot and their dispersion (SEM) by red bars. (B-E) Impact of perceptual variables, including (B) pleasantness, (C) emotional strength, (D) intensity, (E) familiarity, and F) perceptual distance between odors on the predicted probability to correctly recognize an odor. (G-I) Impact of descriptive variables, including (G) the number of words in the description, and (H) semantic distance between descriptions on the predicted probability to correctly recognize an odor. The blue lines represent a simplified general trend fitted by generalized linear regression. **p < 0.01 by GLMM.

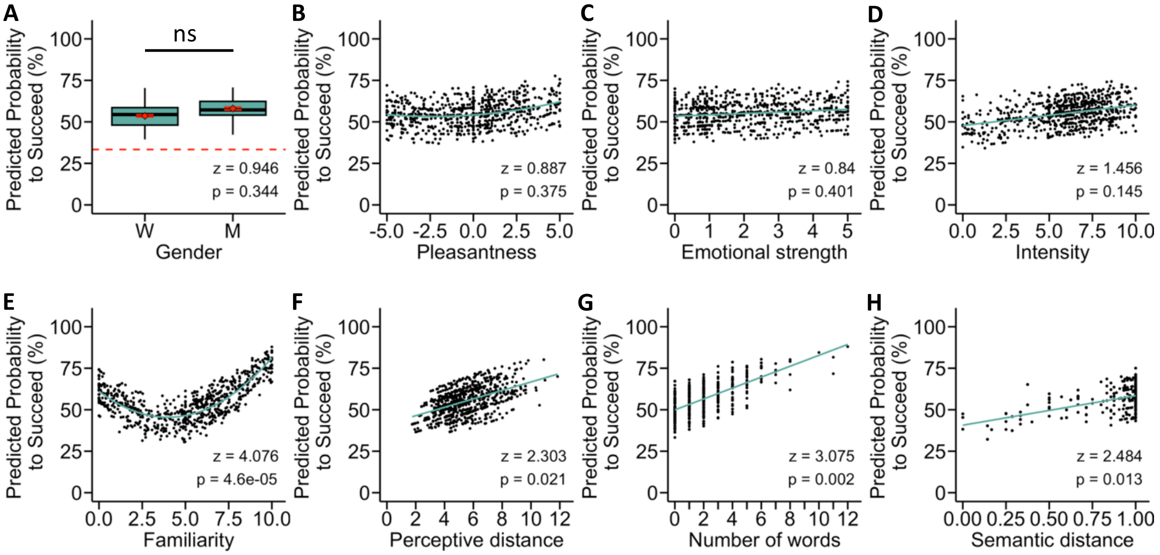

Figure S5. Effects of individual predictors on associative memory align with machine learning results.

(A) Impact of gender (binary: women vs. men) on the predicted probability of associative retrieval. The red dashed line corresponds to the chance level. The distribution of the predicted probabilites is displayed using boxplots in black. Estimated means are represented by a red dot and their dispersion (SEM) by red bars. (B-E) Impact of perceptual variables, including (B) pleasantness, (C) emotional strength, (D) intensity, (E) familiarity, and F) perceptual distance between odors on the predicted probability of associative retrieval. (G-I) Impact of descriptive variables, including (G) the number of words in the description, and (H) semantic distance between descriptions on the predicted probability of associative retrieval. The green lines represent a simplified general trend fitted by generalized linear regression. ns: no significant by GLMM.

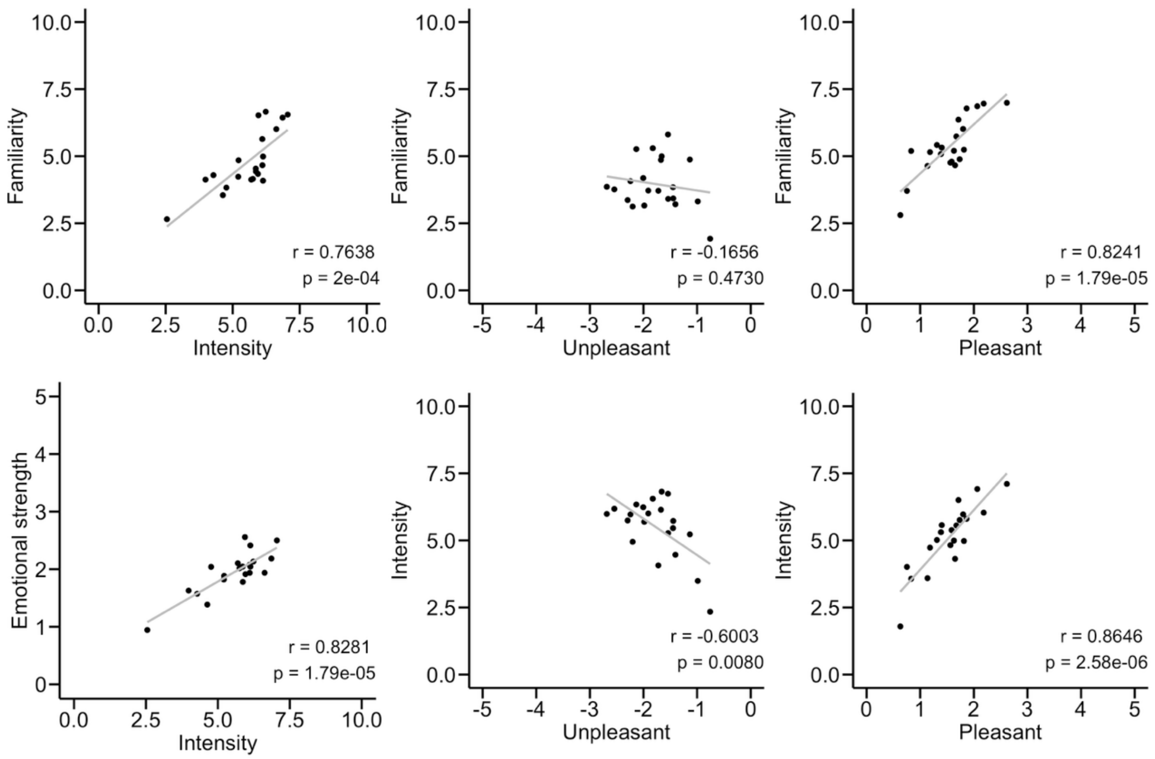

Figure S6. Perceptual ratings are correlated with each other.
Each point represents the average ratings of a single odor. P-values are represented with Holm corrections.

Table S1. Dispersion of ratings across dimensions.

Rating parameters	Theoretical SD	Mean SD_{target}	$Ratio_{target}$ (%)	Mean SD_{hit}	$Ratio_{hit}$ (%)
Pleasantness	5	2.38	47.60	2.39	47.80
Emotional strength	2.5	1.53	61.20	1.52	60.80
Intensity	5	2.32	46.40	2.26	45.20
Familiarity	5	2.78	55.60	2.72	54.40

Theoretical SD refers to the maximum possible standard deviation for each rating scale, based on its defined range (e.g., 5 for scales from –5 to +5 and 0 to 10, and 2.5 for scales from 0 to 5). Mean SD_{target} and Mean SD_{hit} correspond to the average standard deviation computed across all odors in the target and hit sets, respectively. The ratio values express the average standard deviation as a percentage of the theoretical maximum, indicating how dispersed participants' ratings were for each dimension.

Table S2. Participant Demographics Across Studies.

Study	Women	Men	Total	Age Range (years)
Saive et al., 2014b	13	12	25	18-26
Saive et al., 2015	15	8	23	19-26
Plailly et al., 2019	23	8	31	19-29
Unpublished study	14	13	27	18-27

Table S3. List of odors used in the 4 studies.

Odorant	Source
2-heptanone[a]	Sigma-Aldrich®, Saint-Louis, MS, USA
9-decen-1-ol[a]	Sigma-Aldrich
Allyl amyl glycolate[b]	Sigma-Aldrich
Basilic (comores)	Créations aromatiques, Neuilly-Sur-Seine, France
Birch oil	Sigma-Aldrich
Butanol	Sigma-Aldrich
Calone®[b]	Firmenich SA, Genève, Suisse
Carrot	Givaudan, Vernier, Suisse
Citronellol	Sigma-Aldrich
Cis-3-hexenyl salicylate	Sigma-Aldrich
Dihydromyrcenol	Sigma-Aldrich
Ethyl acetyl acetate	Sigma-Aldrich
Linalyl acetate	Sigma-Aldrich
Methyl octine carbonate	Sigma-Aldrich
Musk	Lenoir, Provence, France
Rose oxide	Sigma-Aldrich
Rosemarel®	IFF, New York, NY, USA
Stemone®	Givaudan
Styrallyl acetate[b]	Sigma-Aldrich
Tobacco	Givaudan
Tomato[a]	Givaudan

a, used in Saive et al., 2015 and Plailly et al., 2019
b, used in Saive et al., 2014 and Saive et al., unpublished.
